# Translation, Cross-Cultural Adaptation, Validation and Reliability of the Northwestern Dysphagia Patient Check Sheet (NDPCS) in Iran

**Published:** 2018-03

**Authors:** Jalal Bakhtiyari, Masoomeh Salmani, Reyhaneh Noruzi, Payam Sarraf, Ebrahim Barzegar, Majid Mirmohammadkhani

**Affiliations:** 1 *Neuromuscular Rehabilitation Research Center, Semnan University of Medical Science, Semnan, Iran. *; 2 *Department of Neurology, School of Medicine, Iranian Center of Neurological Researches, Tehran University of Medical Sciences. Tehran, Iran.*; 3 *Department of Speech Therapy, University of Social Welfare and Rehabilitation Sciences, Tehran, Iran.*; 4 *Research Center of Physiology and Department of Community Medicine, Semnan University of Medical Science, Semnan, Iran.*

**Keywords:** Dysphagia, Screening test, Validity, Reliability

## Abstract

**Introduction:**

Speech and language therapists (SLTs) require proper tools to detect dysphagia in the early stages. One of these screening tools is the Northwestern Dysphagia Patient Check Sheet (NDPCS). However, this tool needs to be adapted, validated, and shown to be reliable for the Persian culture. The aim of the present study was to report the validity and reliability of the Persian NDPCS (P-NDPCS).

**Materials and Methods:**

The NDPCS has 28 items and five sections. Beaton’s guidelines were followed in terms of the translation process. To report the content validity index (CVI) and the content validity ratio (CVR), eight SLTs experienced in swallowing disorders examined the content and face validities of the P-NDPCS in terms of the quality of translation, fluency, understandability, and the cultural context. In total, 140 patients with neurogenic and mechanical dysphagia were evaluated using the P-NDPCS. Internal consistency reliability was investigated using the Kuder–Richardson formula 20. The interclass correlation coefficient (ICC) was used for test-retest reliability.

**Results:**

The P-NDPCS preserved the 28 items and the five categories of the original version. However, semantic and food adjustments were applied due to cultural differences. The scoring system was changed from safe/unsafe to yes/no for four subsections and to normal/abnormal for the oromotor section. Food requirements were also changed. The CVR and CVI were both 75%. The P-NDPCS was shown to have good content validity. The internal reliability was 0.95, indicating excellent reliability.

**Conclusion:**

The equivalence between the original version of the NDPCS and the P-NDPCS was preserved. Our findings indicate that the P-NDPCSis a valid and reliable screening tool for the diagnosis of dysphagia in the early phase.

## Introduction

Swallowing is a vital activity. Anyanatomical disorder, such as head and neck cancer, neurological disorders arising from a stroke, or progressive neurological diseases can have negative effects on swallowing (1). Dysphagia, or swallowing disorder, has serious consequences, such as aspiration pneumonia, malnutrition, significant weight loss, dehydration, re-hospitalization, and increasing hospital and therapeutic costs due to medical complications(1,2). However, these problems can be reduced by early diagnosis and early intervention. Speech and language therapists (SLTs) are responsible for the evaluation and management of swallowing disorders. To achieve this, they require valid and reliable tools to screen and to diagnose dysphagia in its early stages (1,3).

Screening tools are designed to be rapid, easy to be administered, minimally invasive, and low-risk to the patients (1). There are many different types of dysphagia screening tools, including the Northwestern Dysphagia Patient Check Sheet (NDPCS), Burke dysphagia screening test, timed swallow test, 3-oz water swallow test, Gugging swallowing screen and the Toronto bedside swallowing screening test (TOR-BSST) (4-9). These tests are available in English and need to be adapted for other languages. While all these tools are designed to detect aspiration, the NDPCS has the potential to detect other problems such as “abnormalities in the oral phase” or “delay in the onset of the pharyngeal phase”.

The NDPCS was designed by Logemann, Veis, and Colangelo in 1998 as a method for the screening of dysphagia (4). The NDPCS has five sections (medical history, behavioral, gross motor function, oromotor evaluation, and evaluation of swallowing) and 28 items. Each item is scored as safe (the presence of some items such as patient cooperation or the absence of some items such as dysarthria) or unsafe. The total score is calculated from the total number of unsafe items. Four signs of dysphagia can be recognized by the patient’s score: presence of aspiration, difficulties in the oral phase, delay of the pharyngeal phase, or any difficulties of the pharyngeal phase. The sensitivity and specificity of this test for English people are each above 70% (4). Swallowing is a part of the feeding process, which is a cultural-based activity, and tools therefore cannot be used without cultural adaptation. The cultural adaptation includes a process that starts with translation and back translation, and then requires measurements of validity and reliability.

The NDPCS questionnaire has previously been translated into a Brazilian-Portuguese version (10). However, the authors only translated and made cultural adaptations for the NDPCS, and did not report any data on the validation process. In the Brazilian version, the number of items and sections is equal to the original version, with adjustments made for only one itemin terms of the food and semantic structure. The swallow trial in the original version involves 1 mL thin liquid, 1 mL pudding, and one-quarter of a Lorna Doone cookie (if chewing was possible); however, in the Brazilian version, the swallow trial includes 5 mL of pudding, 3, 5, and 10 mL of water, and half a wafer cookie. Item 16 (facial weakness) of the original version was changed to orofacial tonicity in the Brazilian version (10).

In Iran, dysphagia management is a relatively new field for SLTs. Iranian SLTs do not have access to validated and reliable tools for the early diagnosis of dysphagia. Regarding the positive aspects of the NDPCS and the necessity for cultural-based tools, the purpose of this study was to report the face and content validities, internal consistency, and reliability of a Persian version of the P-NDPCS.

## Materials and Methods

This research was approved by the Human Participants' Ethics Committee of Semnan University of Medical Sciences (Reference number: IR.SEMUMS.REC.1394.53).

*Stage 1: NDPCS translation process*

The first author gained permission before proceeding to the other stages of the study (2010). The general template for cultural adaptation was based on the guideline presented by Beaton et al. (11). One SLT and one English teacher, both of whom were fluent in Persian and English, translated the original NDPCS. An expert panel including three SLTs and one linguist merged the two Persian forms into one, and then the resulting form was handed to a SLT and another English teacher, who independently translated the P-NDPCS form into English. The final step was to reach agreement between these two forms and the original NDPCS in the same expert panel. Any differences were resolved by the panel.

*Stage 2: Validity and reliability*

Eight SLTs experienced in dysphagia management determined the content and face validities of the P-NDPCS. The scoring criteria were the quality of translation, fluency, understandability, and the cultural context. The results were the content validity ratio (CVR) and the content validity index (CVI) (12,13).

Participants were recruited from the neurology and ear, nose, and throat (ENT) wards of Tehran and Semnan University hospitals by convenient sampling. The examiner evaluated a total of 140 patients using the P-NDPCS. The main inclusion criterion for all patients was the presence of oropharyngeal dysphagia. To calculate reliability, 20 patients were re-evaluated 7–10 days later using the P-NDPCS.

*Stage 3: Statistical testing*

The validity scores were calculated by measures of central tendency (mean). Patients' scores were entered in the SPSS 20. The internal consistency in reliability of the NDPCS was evaluated using the Kuder–Richardson formula 20. The interclass correlation coefficient (ICC) was used to evaluate the test-retest reliability.

## Results

Demographic data for the 140 patients (age, 50 ± 19 years) included in the present study are displayed in (Table.1).

**Table 1:** Demographic data of participants

		**Etiology**		
		**Neurologic**	**Mechanical**	**Total**
Gender	Male	28	41	69
	Female	31	40	71
	Total	59	81	140

The P-NDPCS maintained the 28 items and the five categories. However, some words needed to be replaced by culturally approved substitutes. Instead of safe and unsafe, the dichotomous scoring for the Persian NDPCS includes Yes/No responses for four subsections (medical history, behavioral, gross motor function, and evaluation of the swallowing) and includes normal/abnormal responses for the oromotor section. Some food adjustments were also made; Lorna Doone cookie and pudding were replaced by Salamat biscuit and Danet desert.

The CVR and CVI were each 75%, based on the Lawshe's method. The eight SLTs scored all items as 75% or above. Therefore, we conclude that the P-NDPCS has good content validity. Face validity was approved by the SLTs. Internal consistency of the P-NDPCS is presented in (Table 2).

**Table 2:** Internal consistency and test-retest reliability of the Persian Northwestern Dysphagia Patient Check Sheet (n=20).

**NDPCS, subtest**	**Number of Items**	**ICC**	**P-Value**
Oral motor testing*	16	0/951	< 0/001
Behavioral and gross motor function**	8	0/899	< 0/001
28 variables***	28	0/956	< 0/001

The correlation coefficient was 0/955 for 28 items according to the Kuder–Richardson formula 20 (Table.3), which means the P-NDPCS has excellent reliability.

**Table 3:** Kuder and Richardson Formula 20 for P-NDPCS (n=140).

**KR-20**	**Number of items**	**NDPCS subtest**
Oral motor testing*	16	0/938
Behavioral and gross motor function**	8	0/820
28 variables***	28	0/955

* Total number of Abnormal/Yes observations during oral motor testing and trial swallows

** Total number of Abnormal/Yes observations on behavioral and gross motor function

*** Total number of Abnormal/Yes observations on 28 items in five sections

## Discussion

Different types of instrument are available to identify dysphagia and to evaluate dysphagic patients (14). However, it is not clear from the literature which instrument is most appropriate and applicable to a specific group of patients. This requires researchers to translate rapid, safe, and non-invasive tools such as the NDPCS, adjust them culturally, and apply them to certain types of population (children, adults, or older people). Healthcare professionals find the results of these kinds of studies useful in choosing the most appropriate method to investigate swallowing problems among patients. Therefore, because of its particular characteristics, the NDPCS was chosen for adaptation to the Iranian culture.

The screening session is usually the first time that the therapist meets the patient, and s/he is not fully aware of the patient's condition and abilities. Thus, any step to examine swallowing should be taken carefully and with caution. In addition, studies are not in agreement about the effect of bolus volume and aspiration (15). However, since the Brazilian paper only described the translation and cultural adaptation phase of the adaptation, it is not obvious what complications the therapist may face in a real situation.

During the process of adaptation, some adjustments are inevitable. In the original version, of the NDPCS, patients were required to swallow 1 mL of thin liquid, 1 mL of pudding, and, if the person could chew, the patient received one-quarter of a Lorna Doone cookie. The Brazilian version of the NDPCS introduced some adjustment in the amount of food required (10). Junior et al. (2013) changed the Lorna Doone cookie to the wafer and they increased the amount of thin liquid and pudding (10).

It is important that the Iranian population is evaluated using its own common foods. In fact, the SLTs should consider the texture and the availability of the food for the evaluation process. Therefore, instead of pudding we used Danet and we replaced the Lorna Doone cookiewith a Salamat biscuit.

In the P-NDPCS, similar to the Brazilian version, the number of items and sections of the original version was preserved. In contrast to the Brazilian version, in which semantic adjustments to item 16 of the questionnaire were made, in the P-NDPCS all items were translated without any specific semantic modification. However, the dichotomous scoring system was changed from safe/unsafe to yes/no and normal/abnormal. These types of scoring are more common among Iranian SLTs.

After the translation and cultural adaptation process, the P-NDPCS underwent the validation process. This requires that the P-NDPCS should be easy to understand and easily applied by clinicians. The findings showed that the P-NDPCS has good content validity and face validity. This makes the P-NDPCS an easy and quick tool to administer in clinical settings. The findings also showed that the P-NDPCS is reliable and has strong test-retest reliability.

Like other studies, this study has some limitations, including the absence of another questionnaire against which to compare the results of the P-NDPCS. Future studies including different groups of participants, such as children, older people, and healthy individuals, may provide different perspectives on the P-NDPCS.

## Conclusion

The P-NDPCS is a valid and reliable tool for dysphagia screening. It is easy, quick, and non-invasive, with minimal risk of aspiration. SLTs can diagnose dysphagia in its early stages using P-NDPCS and can evaluate all swallowing stages. This tool can be used for both research and clinical purposes.
